# Genetic anomalies in fetuses with tetralogy of Fallot by using high-definition chromosomal microarray analysis

**DOI:** 10.1186/s12947-019-0159-x

**Published:** 2019-05-06

**Authors:** Ruan Peng, Ju Zheng, Hong-Ning Xie, Miao He, Mei-Fang Lin

**Affiliations:** Department of Ultrasonic Medicine, Fetal Medical Centre, The First Affiliated Hospital of Sun Yat-sen University, Zhongshan Er Road 58#, Guangzhou, Guangdong China

**Keywords:** Tetralogy of Fallot, 22q11.2 deletion, Chromosomal microarray analysis, Cardiac angle, Copy number variations

## Abstract

**Background:**

The etiology of TOF is complex and the genesis of TOF has been associated with environmental factors and genetic disorders, including chromosomal anomalies, aneuploidies, 22q11.2 deletion and single-gene disease. Previous literatures have shown that a chromosome alteration in about 30% patients with TOF and recently published articles reported that 22q11.2 deletion syndrome accounts for 16% cases with TOF diagnosed postnatally. CMA now is considered as gold standard for detecting genetic anomalies in fetuses with congenital malformations. CMA could detect a 6.6–25% incremental yield of CNVs in CHDs. The aim of this study was to assess the genetic anomalies in fetal tetralogy of Fallot (TOF) by using high-definition CMA.

**Methods:**

This retrospective study reviewed all the fetuses diagnosed with TOF between 2013 and 2018. Prenatal ultrasongraphic findings, including cardiac angle, and the findings of CMA using Affymetrix CytoScan HD array were collected.

**Results:**

Ninety-six fetuses with TOF and known genetic results were enrolled. Right aortic arch was the most common associated anomalies (22.9%). One fetus with trisomy 18, one with 46, XX, t (7;10)(q36;q22), one with 47, XYY and five with trisomy 21 were identified. Clinically significant CNVs occurred in 6.8% and uncertain significant CNVs in 3.4% fetal TOF with normal karyotype. A total of four cases with 22q11.2 microdeletion and two fetuses with Yq11.223q11.23 microduplication have been identified. Genetic anomalies, including chromosomal aberrations and pathogenic CNVs, were significantly higher in the TOF with extracardiac anomaly group than in the TOF without extracardiac anomaly group (*P* = 0.005). Abnormal cardiac angle was noticed in 24.0% fetal TOF. Genetic anomalies were more common in the TOF with abnormal cardiac angle than with normal cardiac angle (*P* = 0.001). On the other hand, abnormal cardiac angle was noticed in 64.3% fetal TOF with genetic anomalies while abnormal cardiac angle occurred in 17.1% fetal TOF with normal genetic results (*P* = 0.001).

**Conclusions:**

Genetic testing should be offered, specially using microarray analysis, for the fetal TOF with abnormal cardiac angle or extracardiac defects.

## Background

Fetal tetralogy of Fallot (TOF) and its variants comprise ventricular septal defect, overriding aorta and outflow obstruction of right ventricle, with an occurrence of about 8–12% in infants suffering with congenital heart diseases (CHDs) [1, 2]. A combination of anterocephalad deviation of the outlet septum and abnormal septoparietal trabeculations is now accepted as the hallmark of TOF [3]. The etiology of TOF is complex and the genesis of TOF has been associated with environmental factors and genetic disorders, including chromosomal anomalies, aneuploidies, 22q11.2 deletion and single-gene disease. Previous literatures have shown that a chromosome alteration in about 30% patients with TOF [4] and recently published articles reported that 22q11.2 deletion syndrome accounts for 16% cases with TOF diagnosed postnatally [5].

Chromosomal microarray analysis (CMA) was recently used to detect microdeletions and microduplications prenatally, which was called as copy number variations (CNVs), aiming to exclude fetuses with genetic syndrome. CMA now is considered as gold standard for detecting genetic anomalies in fetuses with congenital malformations. CMA could detect a 6.6–25% incremental yield of CNVs in CHDs [6–8] and subchromosomal rearrangements could interpret the molecular genesis of heart defects. TOF usually has a favor prognosis after operation, if genetic syndrome could be excluded. It is very important to provide information on the association of fetal TOF and genetic anomalies when prenatal counseling. However, the reports on the relationship between the fetal TOF and genetic anomalies, including chromosomal aberrations and pathogenic CNVs using CMA, are limited.

The aim of this study is to analyze the genetic findings in fetal TOF, and to assess that the association between coexisting anomalies and genetic anomalies, including chromosomal aberrations, 22q11.2 deletion and pathogenic CNVs or not.

## Material and methods

**FIG. 1:**
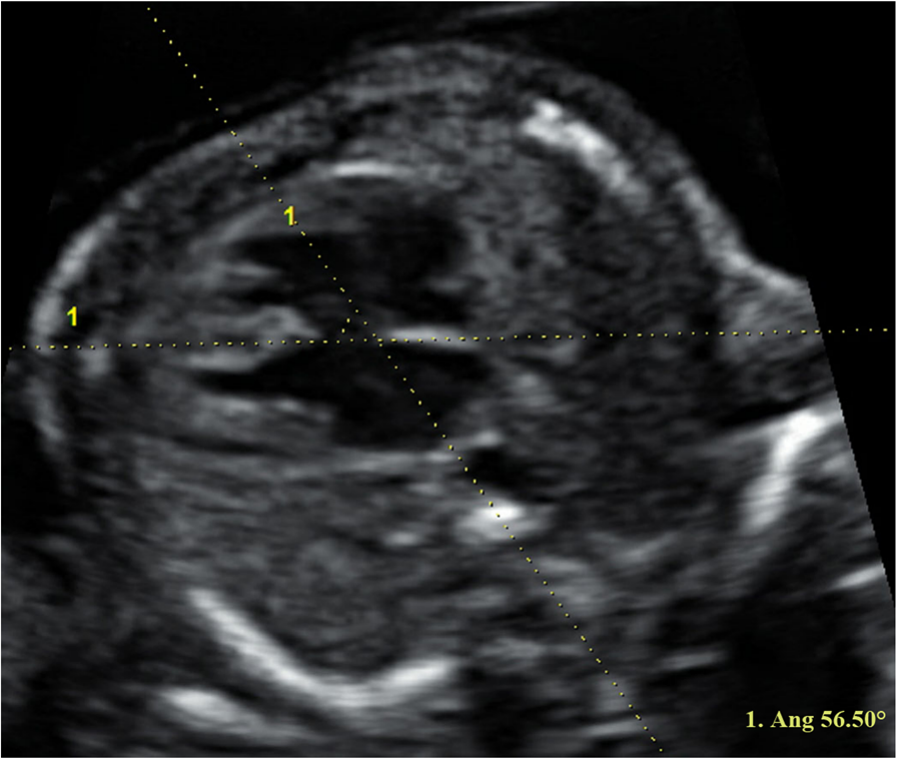
Two dimensional image showing measurement of the cardiac angle in a fetus with tetralogy of Fallot at end systole of the cardiac cycle. Chromosomal microarray analysis revealed that no microdeletion or microduplication was identified in this case and the fetus was delivered at 36 gestational weeks

This study reviewed all the fetuses that were diagnosed with TOF prenatally between 2013 and 2018 in our institution. Prenatal ultrasonographic presentations of TOF are as follows: ventricular septal defect, right ventricular outflow obstruction and an overriding aorta. In this study, three major types of fetal TOF were enrolled, including TOF with pulmonary stenosis, TOF with pulmonary atresia and TOF with an absent pulmonary valve syndrome. A detailed ultrasound screening, including evaluation of intracardiac anatomy and extracardiac structure, was performed in each case. After two dimensional echocardiography examinations, fetal cardiac volumes were acquired with spatiotemporal image correlation (STIC) through the thorax and stored. Fetal cardiac angles were measured by the operator who had no knowledge of the genetic results. The four-chamber view, which showed the interventricular septum, body of the spine and the sternum clearly, was chosen for measurement. Cardiac angle was defined as the angle between the line going through the sternum and the body of the spine and the line tracing the ventricular septum (Fig. 1). Specially, the line goes through the sternum and the body of the spine should bisect the thorax into two equal parts. We made measurements of the cardiac angle at the end of systole period when the mitral valve and the tricuspid valve was closed. A frozen frame from real time images or stored volume data sets could be used for measurement of the cardiac angle. If cardiac angle was within the range 45 ± 20°, it would be considered as normal [9]. A detailed pre-test and post-test counseling was provided in all participants and written informed consents were obtained in all patients. The Ethics Committee of the institution has approved this study. According to the coexisting extracardiac defects, fetuses with TOF were divided into two groups: one group with extracardiac defects (TOF-extra) and the other group without extracardiac defects (TOF-no extra). According to the cardiac angle, fetal TOF was divided into TOF with normal cardiac angle (TOF-CAn) group and TOF with abnormal cardiac angle group (TOF-CAab).

Prenatal samples were processed by microarray-CGH (comparative genomic hybridization) and SNP array (single-nucleotide polymorphism) using Affymetrix CytoScan HD array platform (Affymetrix Inc., Santa Clara, CA, USA) for whole genome-wide analysis. Fetal DNA from cultured amnion or cord blood cells was analyzed for detection of copy number gains or losses. Microarray analysis was performed with the Affymetrix Chromosome Analysis Suite (ChAS) software. The interpretation of copy number gains or losses was performed as follows: First, the detected CNVs were compared with our own database. Then, public available databases, for example, DGV, OMIM, NCBI Gene, DECIPHER, ISCA, CHDWiki, PubMed and so on, were compared. Published literatures were also reviewed when necessary. The detected CNVs were classified into benign CNVs, CNVs of unclear significance (VOUS) and pathological CNVs. Clinically significant CNVs are defined as those that are de novo, relatively large size, and/or contained clinically relevant genes or are associated with well-established syndromes [10]. Furthermore, all CNVs were confirmed with quantitative fluorescent Polymerase Chain Reaction (QF-PCR) or fluorescence in situ hybridization (FISH) when necessary.

Fetal cardiac angle, ultrasonographic findings, genetic results and perinatal outcomes were obtained and recorded. Postnatal echocardiography, surgery or autopsy was obtained in almost all cases.

Mann-Whitney U test was used to compare continuous variables between different groups. Chi-square test or Fisher’s exact test was performed for comparisons of categorical variables between different groups. The software package of SPSS version 22.0 (SPSS Inc., Chicago, IL, USA) was used for statistical analysis. *P* value of <0.05 was considered as significant.

## Results

One hundred and twenty-four fetuses were suspected with TOF prenatally between 2013 and 2018, with a median gestational age of 23^+ 1^ weeks. Genetic data or postnatal data could not be obtained in twenty-six cases. Two suspected cases were confirmed as ventricular septal defect by postnatal echocardiography and so these two cases were excluded. A total of ninety-six confirmed fetuses with TOF were enrolled and genetic results and clinical outcomes were collected in all these 96 fetuses, including 80 TOF with pulmonary stenosis, 14 TOF with pulmonary atresia and two TOF with an pulmonary valve absent syndrome (Table 1). Fetal TOF with extracardiac defects (TOF-extra) were identified in 49 (51.0%, 49/96) cases and TOF without extracardiac defects (TOF-no extra) were identified in 47 (49.0%, 47/96) cases. The associated extracardiac defects in the fetuses with TOF are shown in Table 2. Furthermore, coexisting intracardiac anomalies were noticed in 43.8% (42/96) fetal TOF and right aortic arch was the most common coexisting anomaly, with an incidence rate of 22.9% (22/96). The type, number and frequency of associated cardiac anomalies are listed in Table 3. An absent ductus arteriosus was also seen in fourteen cases (14.6%, 14/96). The median of cardiac angle was 50.3°(range from 26.7°to 81.1°). Normal cardiac angle was detected in 76.0% (73/96) cases and the remaining 24.0% (23/96) cases were identified as an abnormal cardiac angle.

**Table 1:** Demographic characteristics of fetuses with prenatal diagnosis of TOF

Characteristic	Value
Maternal age (years)	30 (19~42)
Gestational age at diagnosis (weeks)	23^+ 1^ (16^+ 2^~31^+ 4^)
TOF type	
TOF with pulmonary stenosis	80 (83.3%)
TOF with pulmonary atresia	14 (14.6%)
TOF with an pulmonary valve absent syndrome	2 (2.1%)
Extracardiac anomaly diagnosed prenatally	49 (51.0%)
Intracardiac defects	42 (43.8%)
Abnormal karyotype	8 (83.3%)
Termination of pregnancy	44 (45.8%)
Selective reduction	14 (14.6%)
Livebirth	35 (36.5%)

Data are reported as median (range) or *n* (%)

**Table 2:** Associated extracardiac defects in fetuses with TOF

Extracardiac defects	*n* (%)
Hypoplasia of the nasal bone	15 (15.6%)
Short long bone	10 (10.4%)
Fetal growth restriction	9 (9.4%)
Single umbilical artery	8 (8.3%)
Hypoplasia of the thymus	6 (6.3%)
Nuchal fold thickening (≥6 mm)	6 (6.3%)
Ventriculomegaly	5 (5.2%)
Heterogeneous echo pattern of liver	4 (4.2%)
Hypoplasia of phalanx	4 (4.2%)
Echogenic bowl	3 (3.1%)
Echogenic kidneys	2 (2.1%)
Club foot	1 (1.0%)
Omphalocele	1 (1.0%)
Low-set ears	1 (1.0%)
Overlapping fingers	1 (1.0%)
Arachnoid cyst	1 (1.0%)
Micrognathia	1 (1.0%)
Ectrodactylia	1 (1.0%)
Cystic hygroma	1 (1.0%)
Strawberry-shaped skull	1 (1.0%)
Long-eyebrows	1 (1.0%)
Cerebellar hypoplasia	1 (1.0%)
Hypoplasia of the lung	1 (1.0%)
Esophago-tracheal fistula	1 (1.0%)
Exencephaly	1 (1.0%)
Hemivertebra	1 (1.0%)

**Table 3:** The type, number and frequency of coexisting cardiac anomalies in fetuses with TOF

Intracardiac anomalies	*n* (%)
Right aortic arch	22 (22.9)
Not present or visualized ductus arteriosus	14 (14.6)
Persistent left superior vena cava	8 (8.3)
Endocardial cushion defect	6 (6.3)
Aberrant right subclavian artery	1(1.0)
Major aortopulmonary collaterals	1(1.0)
Left pulmonary artery sling	1(1.0)
Tricuspid atresia	1(1.0)

Among the 96 fetal TOF with known chromosomal karyotype, eight fetuses were confirmed with chromosomal anomalies, including one with trisomy 18, one with 46, XX, t(7;10)(q36;q22), one with 47, XYY and five with trisomy 21. Chromosomal microarray analysis also verified the chromosomal aberrations in these seven fetuses with unbalanced rearrangement. On microarray, pathogenic CNVs was demonstrated in 6.8% (6/88) fetal TOF with normal karyotype. In our study, a total of four cases with 22q11.2 microdeletion and two fetuses with Yq11.223q11.23 microduplication have been identified. In addition, uncertain significant microdeletions or microduplications were detected in 3.4% (3/88). Benign CNVs also detected in four cases (case 41, case 42, case 72 and case 81). The prenatal ultrasound findings, genetic results and clinical outcomes in these eight fetal TOF with abnormal karyotype and nine with copy variations of uncertain significance or pathogenicity on microarray analysis are shown in Tables 4 and 5. In conclusion, genetic anomalies occurred in 14.6% (14/96) fetal TOF, including chromosomal aberrations and pathogenic CNVs.

**Table 4:** The prenatal ultrasound findings and clinical outcomes in the eight fetuses with TOF and chromosomal abnormalities

Case	Intracardiac anomalies	Extracardiac defects	Fetal karyotype	CMA findings	Pathological significance	Outcome
2	Right aortic arch	Ventricularmegaly	47, XYY	arr[hg]X × 1, Y × 2	Yes	Survival
25	–	Hypoplasia of the nasal bone, short long bone, nuchal fold thickening	47, XX, + 21	arr21q11.2q22.3(15,190,686-48,097,372) × 3	Yes	Selective reduction
26	–	Strawberry-shaped skull, long-eyebrows, overlapping fingers, hypoplasia of the thymus	47, XX, + 18	arr18p11.32q23(136,227-78,013,728) × 3	Yes	TOP
34	Endocardial cushion defect	Ventricularmegaly, hypoplasia of the nasal bone, short lone bone, echogenic bowl	47, XY, + 21	arr21q11.2q22.3(15,190,868-48,097,372) × 3	Yes	TOP
43	–	Ventricularmegaly, hypoplasia of the nasal bone, hypoplasia of the phalanx, short long bone	47, XY, + 21	arr21q11.2q22.3(15,190,868-48,097,372) × 3	Yes	TOP
50	Right aortic arch, ductus arteriosus not visualized	Nuchal fold thickening	47, XY, + 21	arr21q11.2q22.3(15,190,868-48,097,372) × 3	Yes	Survival
88	–	Hypoplasia of the nasal bone, short long bone, nuchal fold thickening, echogenic bowl	47, XY, + 21	arr21q11.2q22.3(15,190,868-48,097,372) × 3	Yes	TOP
93	–	Hypoplasia of the nasal bone, short long bone	46, XX, t(7;10)(q36;q22)	arr[hg] (1–22) ×2, X × 2	–	TOP

*CMA* chromosomal microarray analysis, *TOP* termination of pregnancy

**Table 5:** Characteristic of the fetuses with copy number variants detected by chromosomal microarray analysis among 88 fetuses with TOF and normal karyotype

Case	Intracardiac anomalies	Extracardiac defects	Fetal karyotype	CMA findings	Pathological significance	Outcome
8	Right aortic arch, persistent left superior vena cava	Hypoplasia of the thymus	46, XX	arr22q11.21 (18,916,842-21,798,907) × 1, 2.882 Mb	Yes	TOP
3	Persistent left superior vena cava, aberrant right subclavian artery	–	46, XY	arrYq11.223q11.23 (24,988,143-28,423,925) × 2, 3.436 Mb	Yes	Selective reduction
19	–	Single umbilical artery	46, XX	arr2p24.3 (13,529,731-14,360,751) × 3, 831Kb	VOUS	TOP
35	–	–	46, XY	arrYq11.223q11.23 (24,651,462-28,423,925) × 2, 3.772 Mb	Yes	Survival
46	–	Single umbilical artery, hypoplasia of the nasal bone, heterogeneous echo pattern of the liver	46, XX	arr16p13.3 (1,988,121-2,551,691) × 3, 564Kb	VOUS	IUD
51	–	–	46, XY	arr8p23.3 (1,974,181-2,193,914) × 1, 219Kb; arr8p23.2 (2,202,357-2,730,902) × 3, 529Kb; arr14q11.2 (22,624,119-22,940,347) × 1, 316Kb	VOUS, VOUS, benign	TOP
62	–	Hypoplasia of the thymus, low-set ears	46, XX	arr 22q11.21 (18,916,842-21,465,659) × 1, 2.549 Mb	Yes	Selective reduction
67	Right aortic arch	Nuchal thickening	46, XY	arr 22q11.21 (18,916,842-21,798,907) × 1, 2.882 Mb	Yes	TOP
95	Right aortic arch	Hypoplasia of the thymus	46, XY	arr22q11.21 (18,636,749-21,800,471) × 1, 3.164 Mb	Yes	TOP

*CMA* chromosomal microarray analysis, *TOP* termination of pregnancy, *VOUS* variations of uncertain significance, *IUD* intrauterine death

Excluding fetuses that underwent selective reduction or termination of pregnancies, the overall survival rate was 92.1% (35/38) because three fetuses suffered with intrauterine death. Survival rate was not significantly different between TOF with extracardiac defects (TOF-extra) group and the TOF without extracardiac defects (TOF- no extra) group. Genetic anomalies, including chromosomal aberrations and pathogenic CNVs, were significantly higher in the TOF-extra group than in the TOF-no extra group (*P* = 0.005). However, the incidence rate of intracardiac anomalies, the incidence rate of variations of uncertain significance (VOUS) and cardiac angles were not statistically different between these two groups. More details of these two groups are shown in Table 6. Prevalence rate of genetic anomalies had no significant difference between fetal TOF with intracaridac anomalies (16.7%, 7/42) and fetal TOF without intracardiac anomalies (13.0%, 7/54) (*P* = 0.610).

**Table 6:** Characteristics and genetic results in fetal TOF with extracardiac defects (TOF-Extra) group and in fetal TOF without extracardiac defects (TOF-no Extra) group

Characteristics	TOF-Extra (*N* = 49)	TOF-no Extra (*N* = 47)	*P* value
Maternal age, years	31 (19~42)	31 (21~38)	0.773
Gestational age at diagnosis, weeks	22^+ 6^ (16^+ 2^~30^+ 1^)	23^+ 2^ (18^+ 5^~31^+ 4^)	0.422
Intracardiac anomalies (%)	49.0 (24/49)	38.3 (18/47)	0.292
Genetic anomalies (%)	24.5 (12/49)	4.3 (2/47)	0.005
VOUS (%)	4.1 (2/49)	2.1 (1/47)	1.000
Cardiac angle (°)	50.4 (25.7~73.4)	50.2 (29.2~81.1)	0.613
Abnormal cardiac angle (%)	26.5 (13/49)	21.3 (10/47)	0.547
Survival rate (%)	87.5 (14/16)^a^	95.5 (21/22)^a^	0.562

^a^Cases that underwent selective reduction or termination of pregnancies were excluded. VOUS, variations of uncertain significance

In our data, genetic anomaly was more common in the fetal TOF with abnormal cardiac angle (TOF-CAab) group than in the fetal TOF with normal cardiac angle (TOF-CAn) group, with a rate of 39.1% (9/23) and 6.8% (5/73), respectively (*P* = 0.001). In the other side, abnormal cardiac angle was noticed in 64.3% (9/14) fetal TOF with genetic anomalies while abnormal cardiac angle occurred in 17.1% (14/82) fetal TOF with normal genetic results (*P* = 0.001). Conversely, the difference of the survival rate, the incidence rate of intracardiac anomalies, the incidence rate of extracardiac anomalies and the detection rate of VOUS did not reach statistical significance between the TOF-CAab group and the TOF-CAn group. Prevalence rate of genetic anomalies was not significantly different between TOF with pulmonary atresia (7.1%, 1/14), TOF with pulmonary stenosis (16.3%, 13/80) and TOF with an absent pulmonary valve syndrome (0%, 0/2) (*P* = 0.565). Prenatal ultrasound findings, genetic results with CMA and postnatal outcomes in TOF-CAab group and TOF-CAn group are listed in Table 7.

**Table 7:** Prenatal ultrasound findings, genetic results and postnatal outcomes in fetal TOF with a normal cardiac angle group (TOF-CAn) and in fetal TOF with an abnormal cardiac angle group (TOF-CAab)

Characteristics	TOF-CAn (*N* = 73)	TOF-CAab (*N* = 23)	*P* value
Maternal age, years	30 (20~42)	30 (19~38)	0.619
Gestational age at diagnosis, weeks	23^+ 3^ (16^+ 2^~30^+ 3^)	22^+ 6^ (18^+ 5^~31^+ 4^)	0.955
TOF type			
TOF-pulmonary valve atresia (%)	13.0 (11/73)	14.3 (3/23)	0.695
TOF-pulmonary valve stenosis (%)	82.2 (60/73)	87.0 (20/23)	
TOF-an absent pulmonary valve syndrome (%)	2.7 (2/73)	0 (0/23)	
Intracardiac anomalies (%)	43.8 (32/73)	39.1 (9/23)	0.691
Extracardiac defects (%)	50.7 (37/73)	52.2 (12/23)	0.901
Genetic anomalies (%)	6.8 (5/73)	39.1 (9/23)	0.001
VOUS (%)	1.4 (1/73)	8.7 (2/23)	0.142
Survival rate (%)	85.7 (18/19)^a^	88.9 (17/19)^a^	1.000

^a^Cases that underwent selective reduction or termination of pregnancies were excluded. VOUS, variations of uncertain significance

## Discussion

To the best of our knowledge, our study was one of the largest to evaluate the fetuses with TOF and the associated genetic anomalies. In this study, prenatal ultrasound characteristics, genetic results and clinical outcomes were reviewed and analyzed in 96 fetuses with TOF. TOF was one of the commonest congenital heart disease in cyanosis. Glessner et al. found an increased prevalence of de novo CNVs in cases with conotruncal malformations [11]. Our data showed that pathogenic CNVs occurred in 6.8% fetal TOF and the published article demonstrated that pathogenic subchromosomal arrangements were identified in 5.3% of conotruncal heart defects in our institution [12]. However, the incremental yield of microarray analysis was lower than the meta-analysis study, which reported that an incremental yield of 12% in fetuses with CHDs [13]. So, we do not have sufficient evidence to make a conclusion that pathogenic CNVs are more common in conotruncal heart defects.

Chromosome 22q11.2 deletion syndrome, which is also called as DeGorge Syndrome, is the second most common cause of CHD, with a rate of 0 to 18% in different types of CHDs [4, 14–16]. TOF, interrupted aortic arch, ventriculoseptal defect and truncus arteriosus are the most common cardiac anomalies in patients with 22q11.2 deletion syndrome. Our findings demonstrated that 22q11.2 microdeletion was identified in 4.2% (4/96) fetal TOF. The reported rates of 22q11.2 deletion in TOF were varying from 0 to 18% [4, 16, 17]. A meta-analysis study demonstrated that 18.6% of fetal TOF was complicated with 22q11.2 deletion [18] and TOF with pulmonary stenosis was more frequently associated with major chromosomal anomalies, while 22q11.2 deletion syndrome occurred more common in fetal TOF with pulmonary atresia and TOF with an absent pulmonary valve syndrome [18]. In our series, prevalence rates of chromosomal anomalies and 22q11.2 deletion were not significantly different among TOF with pulmonary atresia, TOF with pulmonary stenosis and TOF with an absent pulmonary valve syndrome. The reason may be that the sample size of TOF with atresia and TOF with an absent pulmonary valve syndrome was relatively small.

A significant proportion of the patients were detected with extracardiac anomalies and coexisting with extracardiac anomaly significantly increased the risk for genetic anomalies. A detailed ultrasound evaluation is recommended to exclude extracardiac anomalies, especially to exclude soft markers and hypoplasia of the thymus, and genetic testing with CMA is suggested when coexisting with extracardiac defects. Just as the recommendations published by the American College of Obstetricians and Gynecologists and the Society for Maternal-Fetal Medicine, the fetuses with one or more major structural abnormalities was suggested to perform invasive prenatal diagnosis using chromosomal microarray analysis [19]. Abnormal cardiac angle may be caused by congenital heart diseases [9], congenital diaphragmatic hernia, [20] abdominal wall defects, [21] occupying lesions such as bronchopulmonary sequestration [22] and so on. Conotruncal malformations may coexist with an abnormal cardiac angle. The study of Zhao demonstrated that the cardiac angle was abnormal in 11.6% out of the 527 cases with CHDs if doing measurement at end systole and they reported that the CHDs with right ventricular volume overload held the highest incidence of abnormal cardiac angle [23]. Abnormal cardiac angle may be caused by the external forces and genetic and molecular markers [24]. The relationship between abnormal cardiac angle and chromosomal anomalies has been reported [25]. Leftward rotation of the fetal cardiac was more severely in fetuses with CHDs and 22q11.2 deletion syndrome when comparing with those with CHDs but no 22q11.2 deletion syndrome [25]. In our cohort, levorotation of the cardiac was noticed in the fetuses with chromosomal aberrations, 22q11.2 deletion syndrome and pathogenic CNVs. The mechanism of leftward rotation in fetuses with 22q11.2 deletion and aneuplodies has been speculated as that was due to the aplasia or hypoplasia of the thymus.

In this study, uncertain significant submicroscopic chromosomal arrangements have been revealed in three cases. In case 19, a 831-Kb microduplication in chromosome 2p24.3 was revealed in the fetus with TOF and single umbilical artery. The gains have not been reported in the normal population. The parents opted for termination of pregnancy because of the cardiac defects. We made a conclusion that the 831-Kb microduplication in this case was VOUS. Case 46 exhibited a 564-Kb microduplication on the band of chromosome 16p13.3 in the fetus with TOF, single umbilical artery, hypoplasia of the nasal bone and heterogeneous echo pattern of the liver. Unfortunately, the fetus suffered with intrauterine death. The deletion region comprises *TSC2*, *PKD1*, *TBC1D24* and *ABCA3* genes and mutations of these genes are associated with tuberous sclerosis complex, autosomal dominant polycystic kidney disease type 1 (*ADPKD1*) or familial infantile myoclonic epilepsy. A 219-Kb deletion in chromosome 8p23.3, a 529-Kb duplication of chromosomal 8p23.2 and a 316-Kb deletion of chromosome 14q was revealed in case 51. However, ultrasonographic finding of this case was isolated TOF. Phenotype of the reported cases partially overlapping 8p23.3 included hypoplasia of the corpus callosum, delayed speech and language development, intellectual disability, and so on. The deletion region of this case has not been found in DGV, DECIPHER or ISCA. The search for 8p23.2 identified the similar results. The pregnancy was also terminated. In our institution, parents usually choose to terminate the pregnancies with TOF, even though the microdeletions or microduplications are interpreted as VOUS prenatally. The study of Shanshen E aimed to identify potential novel CHD candidate genes and they demonstrated that a high incidence of abnormal genes identified by CMA in CHD patients, including many CNVs of “unknown clinical significance” [26]. Clinical and laboratory evidence is need for confirmation the pathogenicity of CNVs of VOUS.

There were several limitations of this study. Firstly, the retrospective design of this study limited that all the cardiac angles were measured retrospectively. Secondly, two of the three fetuses with TOF and uncertain significant CNVs opted for termination of pregnancies. Phenotype after delivery and long-term prognosis, such as cognitive performance or motor development, were not available in these two cases.

## Conclusions

In summary, the findings of this study demonstrated that clinically significant microdeletions or microduplications occurred in 6.8% and uncertain significant CNVs was detected in 3.4% fetal TOF with normal karyotype. Genetic anomalies were more commonly associated with extracardiac anomalies and abnormal cardiac angle. A detailed ultrasonographic assessment, including intracardiac and extracardiac structures, should be performed in the fetus with TOF. We would therefore advocate that genetic testing with chromosomal microarray analysis should be recommended and offered when a fetal TOF coexists with extracardiac defects or abnormal cardiac angle.

## Abbreviations

CHDs: congenital heart diseases
CMA: chromosomal microarray analysis
CNVs: copy number variations
STIC: spatiotemporal image correlation
TOF: Tetralogy of Fallot
VOUS: variations of uncertain significance
